# Different combination strategies for prophylaxis of venous thromboembolism in patients: A prospective multicenter randomized controlled study

**DOI:** 10.1038/s41598-018-25274-2

**Published:** 2018-05-29

**Authors:** Cui-Qin Sang, Na Zhao, Jian Zhang, Shu-Zhen Wang, Shu-Li Guo, Shu-Hong Li, Ying Jiang, Bin Li, Jian-Liu Wang, Lei Song, Jian-Jun Zhai, Zhen-Yu Zhang

**Affiliations:** 1 Department of Obstetrics and Gynecology, Beijing Chao-Yang Hospital, Capital Medical University, Beijing, 100020 China; 2 Department of Obstetrics and Gynecology, Beijing Anzhen Hospital, Capital Medical University, Beijing, 100029 China; 3 Department of Obstetrics and Gynecology, Peking University People’s Hospital, Beijing, 100044 China; 4 Department of Obstetrics and Gynecology, Chinese PLA General Hospital, Beijing, 100853 China; 5 Department of Obstetrics and Gynecology, Beijing Tongren Hospital, Capital Medical University, Beijing, 100730 China

## Abstract

The aim was to evaluate the efficacy and safety of different combination strategies for prophylaxis of venous thromboembolism (VTE) after gynecologic surgery in patients at different levels of risk. This was a prospective multicenter randomized controlled study, in which 625 women who would undergo pelvic surgery for gynecologic diseases were stratified into three risk groups and then randomized into four groups to receive graduated compression stockings (GCS) alone (group A), GCS + low molecular weight heparin (LMWH) (group B), GCS + intermittent pneumatic compression (IPC) (group C), and GCS + IPC + LMWH (group C), respectively. The overall incidence of DVT was 5.1%. Group A had the highest incidence of DVT (8.8%), followed by group C (5.2%), group B (3.8%), and group D (2.6%). There was a significant difference in the incidence of DVT between groups A and D. The incidence of DVT was significantly lower in LMWH-treated patients (group B + group D) than in non-LMWH-treated patients (group A + group C). In conclusion, combination prophylaxis, especially LMWH-containing strategies, is better than monoprophylaxis in reducing VTE after gynecologic surgery. Risk-stratified prophylactic strategies should be implemented in patients undergoing gynecologic surgery, with LMWH-containing strategies being recommended for high-risk and very-high-risk patients.

## Introduction

Venous thromboembolism (VTE), manifesting mainly as deep venous thrombosis (DVT) and pulmonary embolism (PE), is a common serious complication of gynecologic surgery^1^. Epidemiological studies from our team and other groups show that the incidence of DVT following gynecologic surgery ranges from 6.2% to 37.9%^2–4^. Although the true incidence of PE in patients after gynecologic surgery is currently unknown, it has been reported that 0.3–4.4% of patients undergoing thromboembolic prophylaxis after gynecologic oncology surgery developed PE^5,6^. PE is the leading cause of mortality following gynecologic surgery, with a mortality rate as high as 40%^2^. Since the majority of patients with fatal PE often die within 30 min following the appearance of symptoms, there is no chance of thrombolytic therapy or surgery. Therefore, the prevention of VTE is of particular importance.

Many interventions and strategies are currently available to reduce the risk of VTE, such as preoperative pharmacologic prophylaxis and mechanical prophylaxis^7^. Heparin, including unfractioned heparin and low molecular weight heparin (LMWH), is the most commonly used pharmacologic agent for the prophylaxis of VTE. Many clinical trials indicate that heparin is a reliable method to decrease VTE in postoperative patients^7,8^. However, high-dose heparin is associated with a risk of perioperative bleeding, while low-dose heparin was found to be inefficient in high-risk patients with gynecologic cancer^9^. Mechanical prophylaxis, including passive methods such as graduated compression stockings (GCS) and active methods such as intermittent pneumatic compression (IPC), can avoid the risk of bleeding associated with heparin usage; however, these methods are insufficient in reducing VTE in high-risk patients or in critically ill patients^10–12^. Thus, more intense prophylactic strategies are required for high-risk patients.

Compared with either pharmacologic or mechanical prophylaxis alone, dual prophylaxis has biological plausibility in further reducing postoperative VTE in high-risk patients^7^. A systematical review indicates that dual prophylaxis is superior to monoprophylaxis in reducing postoperative VTE, even in high-risk patients with gynecologic cancer^13^. The American College of Chest Physicians has recommended dual prophylaxis for patients at high risk for postoperative VTE^7,14^. Consistent with these, in patients after gynecologic surgery for malignancies, the efficacy of mechanical prophylaxis in reducing postoperative VTE can be improved significantly when a pharmacologic prophylactic method was added^11,15^. Our previous study also showed that the combination of two mechanical prophylactic methods (GCS + IPC) was better than mechanical prophylaxis alone (GCS) in preventing VTE in high-risk patients after gynecologic surgery^16^. Combination prophylactic strategies have been recommended by some researchers for patients at very high risk for VTE after gynecologic surgery^17^.

Despite many previous studies on dual prophylaxis for postoperative VTE prevention, the data obtained from gynecologic patients are still limited and there are currently very few large prospective randomized controlled studies. Furthermore, previous studies rarely stratified the risk of VTE among patients to identify the optimal strategy for patients at different levels of risk for VTE after gynecologic surgery. In this prospective multicenter randomized controlled study, we compared the efficacy and safety of GCS alone, GCS + LMWH, GCS + IPC, and GCS + IPC + LMWH in preventing VTE among patients who were stratified to have different levels of risk for VTE after gynecologic surgery.

## Materials and Methods

### Patients

This multicenter randomized controlled trial was sponsored by Beijing Chaoyang Hospital of Capital Medical University and performed from June 2011 to December 2015 at five tertiary referral centers, including Beijing Chaoyang Hospital of Capital Medical University, Beijing Anzhen Hospital of Capital Medical University, The PLA General Hospital, Peking University People’s Hospital, and Beijing Tongren Hospital of Capital Medical University. The study was approved by the Ethics Committee of Beijing Chao-Yang Hospital, Capital Medical University (No. 10-Ke-42). All participants provided written informed consent before enrollment and the study was performed in accordance with the Helsinki II declaration.

Women who would undergo pelvic surgery for gynecologic diseases at the above-mentioned centers were initially assessed for eligibility. Inclusion criteria were: (1) age >18 years; (2) carrying ≥1 risk factor for postoperative VTE (see below for VTE risk stratification); (3) not taking any prophylactic measures before enrollment; and (4) willing to sign a written informed consent form. Gynecologic diseases may be malignant or benign. Malignant diseases included malignancies of the ovary, uterine body, uterine cervix, vulva, and other parts of the pelvis. Benign diseases included uterine myoma, uterine adenomyoma, ovarian benign tumors, pelvic floor prolapsed, and others such as hydrosalpinx, fallopian tube abscess, encapsulated effusion, and mesosalpinx cyst. Exclusion criteria included: (1) preoperative thrombophlebitis or PE; (2) preoperative acute lower extremity venous thrombosis; (3) preoperative thrombocytopenia (platelet count <100 × 10^9^/L) or coagulation disorders; (4) usage of anticoagulant drugs such as aspirin within 1 month; (5) bleeding tendency as revealed by coagulation indexes or previous intracranial or gastrointestinal bleeding; (6) congestive heart failure or pulmonary edema; (7) serious leg abnormalities (such as dermatitis, gangrene, or recent skin grafting), severe lower limb vascular atherosclerosis, lower limb ischemic vascular disease, or severe leg deformity; and (8) imperception of dorsalis pedis artery pulse.

### VTE risk stratification

VTE risk stratification was performed according to the 2008 American College of Chest Physicians guidelines on VTE prevention^1^ and our previous studies on risk factors for VTE^4,18^. Risk factors analyzed included age ≥50 years, hypertension, previous DVT/PE, thrombophilia, previous cerebral infarction, previous myocardial infarction, lower extremity varices, present surgery for malignancy, laparotomy surgery, operative time ≥3 h, and postoperative bed rest time ≥48 h. Patients with ≤2 risk factors but ≥1 risk factor were deemed to have a moderate level of risk for DVT, those with 3 risk factors deemed to have a high level, and those with ≥4 factors deemed to have a very high level.

### Randomization

Eligible patients were randomized into four groups to receive GCS alone (group A; *n* = 159), GCS + LMWH (group B; *n* = 157), GCS + IPC (group C; *n* = 153), or GCS + IPC + LMWH (group D; *n* = 156). A simple randomization method was adopted for this study. Based on the calculated sample size (see below), 660 consecutive random numbers were selected from a random number table, assigned to each patient, and then divided by four. When the remainder was 0, 1, 2, and 3, the patient was allocated to groups A, B, C, and D, respectively. The patients were included sequentially according to the date of surgery.

### Interventions

#### GCS

Knee-length GCS (Tyco Healthcare, USA) were used as previously described^19^ with some modifications. GCS were properly sized based on the maximum leg circumference as follows: small size for patients with a leg circumference ≤30.5 cm, medium size for those with a leg circumference ≤38.1 cm but >30.5 cm, and large size for those with a leg circumference ≤44.5 cm but >38.1 cm^20^. According to the literature, patients should wear GCS from the time of arrival to the operating room until ambulation^21^. In this study, the patients also wore GCS from the time of arrival to the operating room but were allowed to put GCS off before bedtime when they can move their lower limbs (usually 2 hours after surgery), and put on again on the next morning. The patients put GCS on for ~16 hours each day, during which GCS were put off every 6–8 hours to examine if there were ischemic manifestations or skin impairments.

#### IPC

The Kendall Tyco SCD response compression system (USA), a 6-chamber sequential compression device, was used. The pressure started at the ankle at 45 mmHg and moved towards to the leg at 35 mmHg and the thigh at 30 mmHg. Each compression lasted 11 seconds, and the duration of relaxation was spontaneously adjusted according to the venous filling velocity. The therapy started 30 min before surgery and discontinued while the patient can ambulate^1,16^, generally ~24 hours after surgery.

#### LMWH

Dalteparin (Fragmin Injection, 5000 IU in 0.2 ml solution; Pharmacia, Germany) was used for pharmacologic prophylaxis^22,23^. Periumbilical subcutaneous injection of dalteparin 5000 IU was performed 12 h after surgery, once a day for 5 days^24,25^.

### Measurements

Venous blood samples were collected 7 days before and 3 days after surgery to determine hemoglobin, platelets, and coagulation indexes including prothrombin time, thrombin time, activated partial blood coagulation time, fibrinogen, and D-dimer. Routine hematological and biochemical parameters and routine urine parameters were determined 1 day after surgery.

Screening of VTE was performed within 7 days before and 3–5 days after surgery. Initially, color Doppler ultrasound imaging of lower extremities was performed by an experienced professional using an LEGIQ E9 color Doppler system with the probe frequency set at 8.4–9 MHz^26^. If VTE was found or suspected, lower limb venography was performed to confirm the presence of DVT, and computed tomography pulmonary angiography (CTPA) was performed with the Brilliance iCT system (model# 728306, Philips Medical Systems, USA) to detect if PE was present. Once DVT or PE was diagnosed or suspected, a joint consultation involving cardiovascular and respiratory clinicians was launched to decide the treatment regimen.

### Safety evaluation

Safety evaluation was performed as previously described^27,28^. All adverse events reported by participants were recorded by investigators at various centers, and the principal investigator judged whether to discontinue the treatment or adopt clinical interventions. For bleeding events, the location and amount of bleeding, the amount of transfusion, and the transfused blood components were recorded. The amount of bleeding was estimated as previously described^29^.

Bleeding events were classified as major and minor hemorrhagic events. Major hemorrhagic events included life-threatening bleeding, bleeding occurring in major organs or locations, non-surgical site bleeding associated with a decrease in hemoglobin level by ≥20 g/L or requiring transfusion of two or more units of whole blood, surgical site bleeding requiring further interventions such as a second open or endoscopic surgery and vaginal compression therapy, and surgical site bleeding resulting in instable hemodynamics or a decrease in hemoglobin level by ≥20 g/L. Once major bleeding was found, LMWH would be discontinued immediately.

Minor bleeding was defined as all other bleeding events that did not fall under the categories of major bleeding, such as surgical field bleeding, injection site bruising, drainage port bleeding, and vaginal stump bleeding. For minor superficial bleeding, such as injection site bruising and drainage port bleeding, LMWH was not discontinued. For other minor bleeding events including deep bleeding and wound hematoma, LMWH was discontinued.

Heparin-induced thrombocytopenia was diagnosed when a platelet count ≤50 × 10^9^/L or <50% of the baseline platelet count occurred in patients on heparin therapy, with other causes excluded^30^. Once heparin-induced thrombocytopenia occurred, LMWH would be discontinued immediately.

Lower-limb impairments included skin lesions, ulcers, and limb necrosis.

### Follow-up

Patients who were diagnosed with or suspected of having VTE underwent a joint consultation. Once a diagnosis of PE was made, the patient was referred to the respiratory department for treatment and given LMWH anticoagulant therapy, which was gradually switched to oral warfarin to achieve a stable INR of ~2.0. After 2 to 3 weeks, the patient was discharged, continued to take oral warfarin for 6 months with INR maintained at 2.0 to 3.0, and was followed for 6 months in an outpatient manner.

If a diagnosis of DVT was made, the patient was given anticoagulant therapy under the guidance of a cardiovascular clinician, which was gradually switched to oral warfarin achieve a stable INR of ~2.0. After ~2 weeks, the patient was discharged, continued to take oral warfarin for 3 months with INR maintained at 2.0 to 3.0, and was followed for 6 months in an outpatient manner.

For patients without postoperative VTE, they were discharged 5 to 7 days after surgery and followed for 1 month in an outpatient manner or by telephone.

### Sample size calculation

The sample size of this study was calculated according to the findings of our previous studies on patients at high risk for VTE following gynecologic surgery^16,20^, in which we found that the incidence of VTE was 18% (17/96) in the non-prophylaxis group, 12.5% (14/112) in the GCS alone group, 6% (6/94) in the IPC alone group, 1% in the LMWH alone group, and 4.8% (5/104) in the GCS + IPC group. Based on these incidence rates, we assumed that the incidence of VTE should be <1% in the GCS + LMWH and GCS + IPC + LMWH groups. Thus, a sample size of 150 in each group can provide sufficient statistical power. Adding 10% for dropouts, a final sample size was calculated to be 660. An intention-to-treat analysis was not done.

### Statistical analysis

All statistical analyses were performed using SPSS 23.0 software for Windows (SPSS Inc, Chicago, IL, United States). Numerical data, expressed as mean ± standard deviation, were compared using independent samples *t*-tests between groups. Categorical data, expressed as percentages, were compared by χ^2^ tests if the data followed a normal distribution, and otherwise by nonparametric rank sum tests. Fisher’s exact test was adopted if the sample size in any cell was <40 or if the theoretical frequency was <5. To adjust the potential confounding factors such as operative time, unconditional Logistic regression was used to screen the risk factors for VTE. Odds ratios (ORs) and 95% confidence intervals (CIs) were calculated, and a trend test for the ORs from the fitting model was performed. *P*-values < 0.05 were considered statistically significant.

## Results

### Baseline characteristics

**Figure 1:**
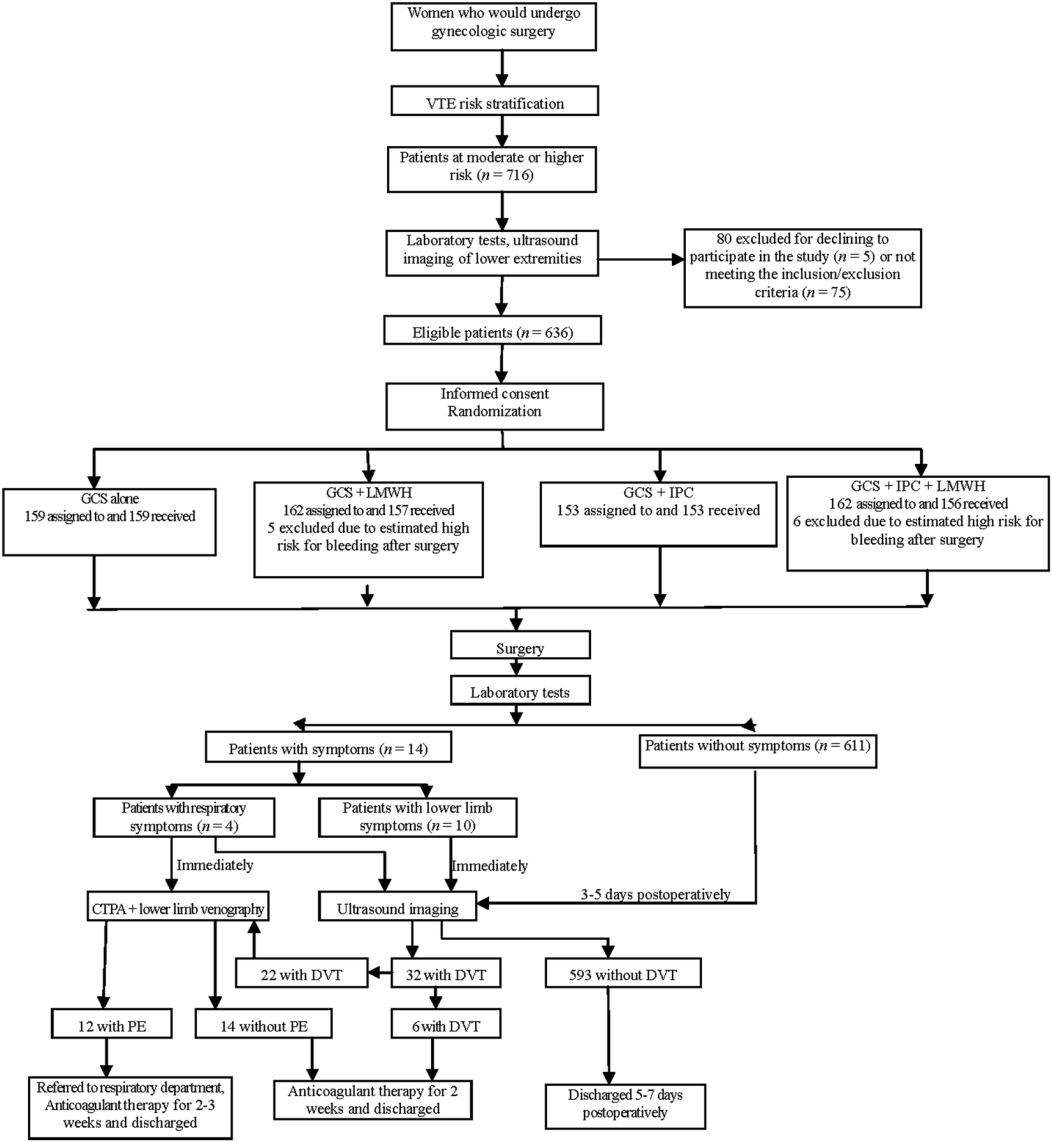
Flow chart of patient enrollment, randomization, and completion of the study.

After risk stratification, 716 patients at a moderate or higher level of risk for VTE were initially identified, of whom 91 were excluded for not meeting the inclusion/exclusion criteria (*n* = 75), refusing to participate in the study (*n* = 5), and estimated high risk for bleeding by surgeons (*n* = 11). Thus, a total of 625 patients were finally included in the study, including 411 moderate-risk patients, 133 high-risk patients, and 81 very-high-risk-patients. Figure 1 shows the flow diagram of patient enrollment, randomization, and completion of the study.

Of the 625 patients finally included, 232 had gynecologic malignancies and 393 had benign gynecologic diseases. They ranged in age from 18 to 86 years, with a mean age of 53.7 ± 10.3 years, and their mean body mass index (BMI) was 24.9 ± 3.7 kg/m^2^. There were no significant differences among the four groups in age, BMI, distribution of diseases including gynecologic malignancies, hypertension, diabetes, lower limb varicosity, respiratory system diseases, cardiovascular diseases, cerebrovascular diseases, digestive system diseases, other malignancies, and anemia, history of thrombosis, usage of anticoagulants or hormonal drugs, or preoperative chemotherapy or radiotherapy (Table 1). Of note, the distribution of patients at different levels of risk for VTE showed no significant difference among the four groups (*P* > 0.05) (Table 2), and there was also no significant difference in the distribution of patients with malignancies among the four groups or among different risk groups (*P* > 0.05) (Table 3).

**Table 1:** Baseline characteristics of the four groups.

	Group A	Group B	Group C	Group D	*P*-value
Age (yr)	54.2 ± 9.4	54.7 ± 11.2	52.6 ± 9.9	53.3 ± 10.5	0.291
BMI (kg/m^2^)	24.8 ± 3.5	24.8 ± 3.6	24.8 ± 3.8	25.4 ± 4.1	0.362
History of thrombosis (%)	1.3	1.9	0.7	2.6	0.570
Hypertension (%)	30.8	29.3	30.1	25.6	0.753
Diabetes (%)	17.0	12.1	11.1	16.7	0.315
History of smoking (%)	1.9	1.9	0.0	0.0	0.115
Varicosity (%)	8.8	7.6	5.9	9.6	0.648
Preoperative anticoagulant usage (%)	1.9	2.6	0	0	0.075
Preoperative chemotherapy (%)	3.8	2.6	2.6	6.4	0.230
Heart disease (%)	6.9	5.7	7.2	3.8	0.585
Respiratory diseases (%)	3.8	1.9	3.3	1.3	0.469
Anemia (%)	10.7	9.6	11.8	9.0	0.856
Open surgery (%)	37.1	34.4	38.6	25.6	0.149
Operative time (min)	165.3 ± 78.7	161.7 ± 81.1	196.6 ± 93.7	198.8 ± 95.3	**0.000**
Blood loss (mL)	50 (20–150)	50 (20–145)	60 (30–200)	50 (20–200)	**0.042**
Transfusion (%)	4.4	1.3	5.9	3.2	0.196

**Table 2:** Distribution of patients at different levels of risk for VTE in the four groups.

Group Risk level	A	B	C	D	Overall	χ^2^
Moderate	102	113	95	101	411	
High	35	31	34	33	133	5.383
Very high	22	13	24	22	81	
Overall	159	157	153	156	625	

Note: *P* < 0.05, among the four groups.

**Table 3:** Distribution of patients with malignancies in the four groups based on risk level.

Group Risk level	A	B	C	D	Overall	χ^2^
Moderate	15	22	18	34	89	
High	15	15	20	21	71	6.651
Very high	18	13	23	18	72	
Overall	48	50	61	73	232	

Note: *P* < 0.05, either among the four groups or among the four risk groups.

With regard to surgical situation, there were no significant differences among the four groups in mode of surgery, patient-controlled analgesia, anesthetic techniques, site of infusion, or blood transfusion (Table 1). However, a significant difference was observed in operative time and blood loss. Operative time was significantly longer in groups C and D than in groups A and B (*P* < 0.05), although there were no significant differences between groups A and B or between groups C and D. Blood loss was significantly more in group C than in the other three groups (*P* < 0.05). To exclude the confounding effect of operative time and blood loss, unconditional Logistic regression analysis was performed, which revealed that the difference remained statistically significant after adjusting for operative time (OR = 0.618, 95%CI: 0.437–0.874, *P* = 0.007), but not for blood loss (OR = 1.001, 95%CI: 1.003–1.01, *P* = 0.189).

### Incidence of DVT

Of the 625 patients included, no perioperative death occurred, and 32 developed DVT, including 14 in group A, 6 in group B, 8 in group C, and 4 in group D. Of all cases of DVT, only one (1.7%, 1/58) was proximal, involving the popliteal vein, and the rest were distal, involving the calf muscular vein (75.9%, 44/58), peroneal vein (17.2%, 10/58), and posterior tibial vein (5.2%, 3/58) (Table 4). Of note, six cases developed DVT at different locations, and 26 cases developed bilateral DVT. Main clinical manifestations of DVT included limb pain, swelling and congestion, skin eczema, local tenderness and dysfunction. Symptomatic patients accounted for only 31% (10/32) in this study.

**Table 4:** Location of DVT.

	Group A	Group B	Group C	Group D	Overall
Calf muscular vein	12/19	6/9	7/11	4/5	29/44
Peroneal vein	3/5	1/2	2/3	0	6/10
Posterior tibial vein	1/2	0	1/1	0	2/3
Popliteal vein	1/1	0	0	0	1/1
Overall	14/27	6/11	8/15	4/5	32/58

Note: Six cases developed DVT at different locations, and 26 cases developed bilateral DVT. Data shown are the number of cases/limbs.

The overall incidence of DVT was 5.1% (32/625). As shown in Table 5, group A had the highest incidence of DVT (8.8%, 14/159), followed by group C (5.2%, 8/153), group B (3.8%, 6/157), and group D (2.6%, 4/156). There was a significant difference in the incidence of DVT between groups A and D (χ^2^ = 5.69, *P* < 0.05), but not between any two other groups. Of note, the incidence of DVT was significantly lower in LMWH-treated patients (group B + group D) than in non-LMWH-treated patients (group A + group C) (χ^2^ = 4.78, *P* < 0.05).

**Table 5:** Incidence of DVT in the four groups based on risk level.

Group Risk level	**A**	**B**	**C**	**D**	**Overall**
Moderate	3/102 (2.9%)	1/113 (0.9%)	2/95 (2.1%)	3/101 (3.0%)	9/411 (2.2%)^#^
High	2/35 (5.7%)	2/31 (6.5%)	2/34 (5.9%)	1/33 (3.0%)	7/133 (5.3%)^#^
Very high	9/22 (40.9%)	3/13 (23.1%)	4/24 (16.7%)	0/22 (0%)^**^	16/81 (19.8%)^#^
Overall	14/159 (8.8%)	6/157 (3.8%)	8/153 (5.2%)	4/156 (2.6%)^*^	32/625 (5.1%)

Note: ^*^P < 0.05 vs. group A; ^#^*P* < 0.01 among the three risk groups; ^**^P < 0.01 vs. group A.

With regard to risk stratification, the moderate-risk patients had the lowest incidence of DVT (2.2%, 9/411), followed by the high-risk patients (5.3%, 7/133) and very-high-risk patients (19.8%, 16/81), and there was a significant difference among the three groups (χ^2^ = 42.97, *P* < 0.01). This finding indicates that the incidence of DVT increased with the risk level when the same intervention was given. Of note, 71.9% (23/32) of DVT cases occurred in the high-risk and very-high-risk groups.

The incidence of DVT in moderate-risk patients in different groups ranged from 0.9% to 3.0%, and group B had the lowest incidence (0.9%, 1/113), followed by group C (2.1%, 2/95). In high-risk patients, group D had the lowest incidence (3.0%, 1/33), followed by group A (6.5%, 2/31). In very-high-risk patients, group D had the lowest incidence (0%, 0/22), while group A had the highest incidence (40.9%, 9/22), and there was a significant difference in the incidence of DVT between them (χ^2^ = 8.94, *P* < 0.01).

### Incidence of PE

Of 32 cases of DVT, 26 underwent CTPA, which revealed that 12 had PE and 14 did not have; six did not undergo CTPA due to hypersensitivity to contrast medium (*n* = 2) or patient refusal (*n* = 4). Of 12 cases of PE, no involvement of pulmonary artery trunk occurred, and all affected pulmonary segmental arteries, including 9 cases of multiple bilateral pulmonary embolisms and 3 cases of single unilateral pulmonary embolism. No fatal PE occurred. Four (1/3) cases had apparent symptoms including chest pain (*n* = 2), mild chest distress (*n* = 1), and postoperative syncope (*n* = 1), all of which disappeared with rest.

The overall incidence of PE was 1.9% (12/625). The incidence of PE in groups A-D was 4.4% (7/159), 0.64% (1/158), 1.96% (3/153), and 0.64% (1/156), respectively (Table 6). The incidence of PE in groups B and D was significantly lower than that in group A (*P* < 0.05), but there was no significant difference between group A and group C. Of note, the incidence of PE was significantly lower in LMWH-treated patients (group B + group D) than in non-LMWH-treated patients (group A + group C) (*P* < 0.05).

**Table 6:** Incidence of PE in the four groups based on risk level.

Group Risk level	A	B	C	D	Overall
Moderate	2/102 (2.0%)	1/113 (0.9%)	0/95 (0%)	0/101 (0%)	3/411 (0.73%)
High	1/35 (2.9%)	0/31 (0%)	1/34 (2.9%)	1/33 (3.0%)	3/133 (0.23%)^#^
Very high	4/22 (18.2%)	0/13 (0%)	2/24 (8.3%)	0/22 (0%)^**^	6/81 (7.4%)^#^
Overall	7/159 (4.4%)	1/157 (0.64%)^*^	3/153 (2.0%)	1/156 (0.64%)^*^	12/625 (1.9%)^#^

Note: ^*^P < 0.05 vs. group A; ^#^*P* < 0.01 among the three risk groups; ^**^P < 0.05 vs. group A.

With regard to risk stratification, the moderate-risk patients had the lowest incidence of PE (0.73%, 3/411), followed by the high-risk patients (2.3%, 3/133) and very-high-risk patients (7.4%, 6/81), and there was a significant difference among the three groups (χ^2^ = 16.12, *P* < 0.01). This finding indicates that the incidence of PE increased with the risk grade when the same intervention was given. Of note, 75% (9/12) of PE cases occurred in the high-risk and very-high-risk groups.

The incidence of PE in moderate-risk patients in different groups ranged from 0.0% to 2.0%, and groups C and D had the lowest incidence (0.0% for both). In high-risk patients, group B had the lowest incidence (0.0%, 0/31). In very-high-risk patients, groups B and D had the lowest incidence (0% for both), while group A had the highest incidence (18.2%, 4/22), and there was a significant difference in the incidence of DVT between them (χ^2^ = 4.40, *P* < 0.05).

### Rate of PE in patients with DVT

The rate of PE in patients with DVT showed no significant difference either among the four groups or among the different risk groups (*P* > 0.05) (Table 7), suggesting that the incidence of PE had a similar trend to that of DVT. Overall, the number of cases with DVT and/or PE was lower in groups B and C for moderate-risk patients, in groups B and D for high-risk patients, and in group D for very high-risk patients.

**Table 7:** Incidence of DVT with PE in the four groups based on risk level.

Group Risk level	A	B	C	D	Overall
Moderate	2/3	1/1	0/2	0/3	3/9
High	1/2	0/2	1/2	1/1	3/7
Very high	4/9	0/3	2/4	0/0	6/16#5
Overall	7/14	1/6	3/8	1/4	12/32

### Adverse events

Table 8 lists the bleeding events observed within 7 days after surgery in the four groups. No major bleeding or heparin-induced thrombocytopenia occurred in this study. Compared with groups A and C, the number of mild bleeding events significantly increased in groups B and D (*P* < 0.01). These mild bleeding events mostly occurred following 2–3 injections of LMWH. After LMWH was discontinued, bleeding stopped and surgical hemostasis was not required. A total of 27 patients discontinued LMWH, but transfusion or hemostasis was not required.

**Table 8:** Bleeding events observed within 7 days after surgery in the four groups.

	Group A	Group B	Group C	Group D
Injection site bruising		13		13
Wound hematoma	0	8	0	6
Drainage port bleeding	0	0	2	3
Vaginal bleeding	0	1	0	5
Pelvic bleeding	2	4	1	0
Hematuria	0	1	0	1
Bleeding of unknown cause	0	1	0	0
Overall	2	28	3	28

During the follow-up period, four cases sought treatment for minor vaginal bleeding, including 1 case in group A, 1 case in group C, and 2 cases in group D. Since the two cases in group D did not develop vaginal bleeding within 7 days after surgery, their bleeding may not be caused by LMWH usage, but may be associated with poor wound healing secondary to local inflammation or suture loosening. After anti-inflammatory treatment, bleeding stopped. In addition, vaginal stump bleeding with an amount like that of menstrual bleeding occurred in a patient with PE 12 days after surgery and a patient with DVT 14 days after surgery, both of whom belonged to group A. Warfarin was discontinued in both cases until bleeding stopped, and then LMWH+ warfarin was restarted.

No lower-limb impairments occurred throughout the study period.

## Discussion

Three primary factors predisposing to thrombosis (Virchow’s triad) are: (1) endothelial injury; (2) stasis or turbulence of blood flow; and (3) hypercoagulability of blood^31^. Directing against these predisposing factors, many prophylactic strategies have been developed to prevent postoperative VTE^7^. Since GCS, IPC, and LMWH are relatively commonly used strategies, the efficacy and safety of their combination in three different ways in preventing VTE after gynecologic surgery were evaluated in the present study.

### Technical details for prophylactic strategies used

Both GCS and IPC are mechanical prophylactic methods, and they can increase venous return, reduce venous distention, and thus prevent venous stasis that contributes to an increased risk of VTE^7^. In this study, IPC was performed using a commercial device as previously described^1,16^, while some modifications were made to the use of GCS. Since knee-length GCS are as effective as thigh-length ones, more comfortable to wear, and easier to apply^19^, the former was used in this study. Although the use of GCS for the entire day was advocated in many previous studies^1,21^, this is associated with an increased risk of pressure ulcer and rarely limb necrosis^19^. Furthermore, improperly fitted stockings may act as a tourniquet to result in venous stasis and increase the risk of VTE^2^. Particularly, the folding and distortion of stockings that easily occur when patients are sleeping can result in the tourniquet effect. For these reasons, we allowed our patients to put GCS off before bedtime once they can move their lower limbs (usually 2 hours after surgery), and put on again on the next morning. This greatly increased patients’ comfort and improved their compliance. As a consequence, the incidence of VTE (8.8%) in the GCS alone group was lower than that reported previously (12.5%)^16^, and no pressure ulcer or limb necrosis occurred throughout the study period.

In this study, we used dalteparin as a pharmacological agent. As an LMWH, dalteparin has the advantages of simplicity, good anticoagulation effects, and low risk of heparin-induced thrombocytopenia^22,23^. Compared to enoxaparin and nadroparin, dalteparin has a shorter plasma elimination half-life (2.8–3.8 h)^32,33^. Thus, once bleeding occurs, discontinuation of dalteparin will result in a more rapid recovery of coagulation function. Due to the combined use of dalteparin with GCS and/or IPC, dalteparin was given later (12 h vs. 2–6 h postoperatively) at a lower dose (5000 U, once daily) in the present study compared to conventional administrations^24,25^. These modifications may explain why no major bleeding events occurred in this study.

### Combination prophylaxis, especially LMWH-containing strategies, is better than monoprophylaxis

Many randomized trials performed in other fields such as urology and general surgery have demonstrated that combination prophylactic strategy is superior to either pharmacologic or mechanical prophylaxis alone in reducing the incidence of postoperative VTE^7^. Despite few trials on gynecologic patients, combination prophylaxis strategy has also been recommended by some researchers for this group of patients, especially those who have a high risk for VTE, such as gynecologic oncology patients after a major surgery^17^. In agreement with previous findings, this study indicated that combination strategies including GCS + IPC (5.2%), GCS + LMWH (3.8%), and GCS + IPC + LMWH (2.6%) were better than GCS alone (8.8%) in reducing the incidence of VTE in patients after gynecologic surgery. Thus, our study provides several additional options for combination prophylaxis of postoperative VTE in gynecologic patients.

A remarkable finding of this study is that LMWH-containing strategies are significantly better than non-LMWH-containing ones in the prophylaxis of postoperative VTE in gynecologic patients. Compared to the combination of two mechanical methods (GCS + IPC), the strategy combining both mechanical and pharmacologic prophylaxis (GCS + LMWH or GCS + IPC + LMWH) exhibited better efficacy. LMWH has been demonstrated to be reliable in decreasing VTE in postoperative patients^7,8^; however, high-dose LMWH may cause perioperative bleeding while low-dose LMWH has insufficient efficacy in high-risk patients^9^. The combination of mechanical prophylaxis with LMWH optimized for dosage and timing (*e.g*., 12 h postoperatively in this study) may overcome these problems. In fact, although LMWH-containing strategies were associated with significantly more bleeding events, all of them were mild and manageable.

### Different prophylactic strategies should be implemented in patients at different levels of risk for VTE

Many risk factors for VTE have currently been identified^4,18^. Based on these risk factors, individual risk assessment can be performed to guide the degree of prophylaxis. This study showed that the incidence of both DVT and PE increased with the risk level even in patients receiving the same intervention, further suggesting the importance of risk stratification to guide the administration of different interventions to patients at different levels of risk. Since the majority of DVT and PE cases were present in the high-risk and very-high-risk groups, the emphasis of prophylaxis should be placed on this group of patients.

An ideal prophylaxis regimen should be tailed to the patient’s thromboembolic risk, thus allowing clinicians to maximize the benefits and minimize the harms^7^. Although all four strategies were effective in the moderate-risk patients, their efficacy still differed. Based on their efficacy, GCS + LMWH and GCS + IPC are recommended for this group of patients although all four strategies are acceptable. When patients are not suitable for the use of anticoagulants, GCS + IPC is recommended to avoid the risk of increased bleeding. For high-risk patients, both LMWH-containing strategies resulted in an acceptable incidence of DVT. From a health-economics point of view, GCS + LMWH is recommended. GCS + IPC + LMWH was associated with the lowest incidence of DVT in the very-high-risk group. Therefore, this strategy is recommended for very-high-risk patients. Of note, GCS alone are not recommended for both high-risk and very-high-risk patients due to its poor preventive effects, which is consistent with the previous finding^34^.

### Limitations

Despite some promising results, there are several limitations to our study. First, the relatively small number of high-risk and very-high-risk patients was underpowered to show statistical differences between groups. In fact, although GCS + LMWH and GCS + IPC exhibited better efficacy than GCS alone in high-risk and very-high-risk patients, no significant difference was observed. Second, 6 of 32 patients with DVT did not undergo CTPA, and this might have resulted in the underestimation of the incidence of PE. Finally, the patients without DVT were followed for 4 weeks in this study; however, it has been reported that DVT may develop 7–12 weeks after surgery^35^. Future studies with larger sample size and longer follow-up periods are needed to carefully address these issues.

## Conclusions

To conclude, this study shows that combination prophylaxis, especially LMWH-containing strategies, is superior to monoprophylaxis in reducing the incidence of VTE after gynecologic surgery. Given the incidence of both DVT and PE increases with the risk level even in patients receiving interventions, risk-stratified prophylactic strategies should be implemented in patients undergoing gynecologic surgery, with LMWH-containing strategies being recommended for high-risk and very-high-risk patients.
